# Comprehensive Identification of Key Genes Involved in Development of Diabetes Mellitus-Related Atherogenesis Using Weighted Gene Correlation Network Analysis

**DOI:** 10.3389/fcvm.2020.580573

**Published:** 2020-10-28

**Authors:** Qi Huang, Guoxiong Deng, Rongguo Wei, Qiaoye Wang, Donghua Zou, Jinru Wei

**Affiliations:** ^1^Department of Cardiology, The Fifth Affiliated Hospital of Guangxi Medical University, Nanning, China; ^2^Department of Cardiology, The First People's Hospital of Nanning, Nanning, China; ^3^Department of Clinical Laboratory, The Fifth Affiliated Hospital of Guangxi Medical University, Nanning, China; ^4^Department of Clinical Laboratory, The First People's Hospital of Nanning, Nanning, China; ^5^Department of Neurology, The Fifth Affiliated Hospital of Guangxi Medical University, Nanning, China; ^6^Department of Neurology, The First People's Hospital of Nanning, Nanning, China

**Keywords:** diabetes mellitus, coronary heart disease, hallmark gene set, WGCNA, atherogenesis

## Abstract

Coronary heart disease (CHD) is common in patients with diabetes mellitus (DM), however, the relevant mechanism remains elusive. The whole blood gene expression profiles of healthy control, patients with DM, patients with DM and CHD (DMCHD) were used to performed weight gene correlation network analysis (WGCNA) to identify the gene modules associated with DM-related atherogenesis. The candidate module was significantly involved in immune- and T cell activity-related biological process. GSEA results suggested that lysosome and apoptosis were enriched in DM and DMCHD samples. The protein-protein-KEGG pathway network may reveal the potential transcriptional regulatory network involving in DM-related atherosclerosis. Nineteen genes (RTKN, DCP1B, PDZD4, CACNA2D2, TSEN54, PVRIG, PLEKHF1, NKG7, ZAP70, NUDCD3, SLAMF6, CCDC107, NAG6, ZDHHC14, EOMES, VIL2, WDR54, DMAP1, and PMPCA) were considered as DM-related atherogenesis genes (DRAGs). The Gene Set Variation Analysis (GSVA) score of the DRAG set gradually increased in the control, DM and DMCHD. ROC curve analysis showed that ZAP70, TSEN54, and PLEKHF1 may be potential blood circulation biomarkers for DMCHD in patients with DM. In conclusion, we identified nineteen hallmark genes involving in DM-related atherogenesis and constructed a potential transcriptional regulatory network involving in DM-related atherosclerosis.

## Introduction

Diabetes mellitus (DM) is a metabolic disorder of the endocrine system, clinically characterized by hyperglycemia as well as alterations in lipids, carbohydrates, and protein metabolism (1, 2). DM can be divided into type 1 and type 2, of which more than 90% of cases are type 2 (3, 4). Thus, only type 2 DM was focused in our present study. The increasing prevalence of type 2 DM means that 430 million people are expected to suffer the condition by 2030 (5, 6). DM renders patients more susceptible to arterial atherosclerosis, which causes cardiovascular disease, especially coronary heart disease (CHD). CHD is the most serious complication of DM and a frequent cause of death among DM patients (7). Thus, elucidating why DM increases risk of CHD is of great importance.

Several studies have suggested that the presence of hypertension, dyslipidemia, and/or obesity in most patients with DM may help explain the increased incidence of CHD, since these factors also increase CHD risk in individuals without DM (8–14). Given that DM is multifactorial and quite heterogeneous (15), a comprehensive examination is required to understand key mechanisms driving development of CHD in patients.

To address this issue, we attempted to identify hub genes or gene sets involved in DM-associated CHD (DMCHD). Using weighted correlation network analysis (WGCNA), we identified 19 DM-related atherogenesis genes (DRAGs). Based on protein-protein interaction and functional enrichment analysis, we constructed a protein-protein pathway network to uncover the potential transcriptional regulatory network involved in DM-related atherosclerosis.

## Materials and Methods

### Data Processing

We downloaded gene expression profiles from the Gene Expression Omnibus (https://www.ncbi.nlm.nih.gov/) (16) to analyze genetic differences between DM and DMCHD. We extracted the data set from the human whole blood gene expression profile of *GSE*34198 based on GPL6947 (17), which included 33 healthy control samples, 34 CHD samples, 15 DM samples and 15 DMCHD samples. In GSE34198, CHD patients refers to patients who eventually develop acute myocardial infarction and DM refers to type 2 diabetes. The clinical information including sex, age, body mass index and smoking status was provided in the Supplementary Table 1. GSE90074 (18) based on GPL6480 was as a validation set and contained human whole blood mononuclear cells gene expression profiles from 33 healthy control samples, 55 CHD samples, 17 DM samples, and 38 DMCHD samples. The *normalize Between Arrays* function in the *limma* package (19) was used to normalize the gene expression profiles. Probes that corresponded to multiple genes were removed. If a gene was detected using multiple probes, the expression level of the gene was calculated by averaging the expression from all the probes.

### Screening Differently Expressed Genes (DEGs) in DM and DMCHD Compared to Healthy Control

To identify the genes involving in DM-related atherogenesis, the DEGs in DM and DMCHD compared to healthy control were screened using limma package. A gene with P adjusted by false discovery rate <0.05 was considered significantly differently expressed.

### WGCNA

It is well-known that DM increases risk of CHD, thus, healthy control, DM, and DMCHD were considered as different stages of DM-related atherosclerosis. According to WGCNA official website (https://horvath.genetics.ucla.edu/html/CoexpressionNetwork/Rpackages/WGCNA/), this method is suitable for identifying gene modules with the certain phenotype (DM-related atherosclerosis). DM increases the risk of CHD is considered the result of multiple molecules working together. WGCNA was used to identify the related gene modules (each module contain multiple molecules). Thus, WGCNA (20) was selected in the present study. The DEGs were subjected to perform a weighted gene expression network in GSE34198 using the WGCNA package (20) in R version 3.5 (https://www.r-project.org/). Candidate power (1 to 20) was used to test the average connectivity degrees of different modules and degrees of independence. If the degree of independence was >0.85, the power value was selected. The *hclust* function was used to cluster samples and check for outliers. Subsequently, a heat map was constructed to correlate gene modules with phenotype and statistical significance. High correlation meant that the genes of the corresponding module tended to be highly correlated with development of CHD in DM patients. In the present study, the module with the most significant positive correlation to DM-related atherogenesis was considered the candidate module.

### Functional Enrichment Analysis of Gene Modules

In order to explore the signaling pathways and biological characteristics of related genes in phenotype-related modules, we conducted Gene Ontology (GO) and Kyoto Encyclopedia of Genes and Genomes (KEGG) pathway enrichment analysis. *The clusterProfiler* package (21) in R were applied to these functional enrichment analysis. In addition, a biological processes network was constructed using the *ClueGO* plug-in (22) in *Cytoscape* (23). GSEA software (https://www.gsea-msigdb.org/gsea/downloads.jsp) was used to analyze gene set enrichment (24) based on the reference gene sets “c5.bp.v7.0. symbols.gmt” and “c2.cp.kegg.v7.0.symbols.gmt” (25). *P* < 0.05 was considered significant.

### Construction of Protein-Protein KEGG Pathway Networks

The STRING database (https://string-db.org/) (26) was used to extract protein-protein interactions between candidate module genes. *Cytoscape* was used to construct a protein-protein network based on KEGG pathways involved in DM-related atherogenesis.

### Identification of DM-Related Atherogenesis Genes (DRAGs) and Assessment of Predictive Potential

In the candidate module based on WGCNA, genes with significance (GS) > 0.2 and module membership (MM) > 0.7 were considered as DM-related atherogenesis genes (DRAGs). The *GSVA* package (27) was used to perform gene set variation analysis (GSVA) to score individual samples against the DRAG set, and the DM-related atherogenesis GSVA score was obtained for each sample. The ability of the DM-related atherogenesis GSVA score to predict DMCHD was evaluated using receiver operating characteristic (ROC) curve analysis with the *pROC* package (28). The aberrant expression of DRAGs were validated using the GSE90074 dataset. DRAGs with high predictive value were considered as biomarkers of DMCHD.

### Cell Subpopulation Analysis

The host's response to DM and DMCHD may also be reflected in blood cell subsets. The webtool xCell (29) (https://xcell.ucsf.edu/) was used to perform cell type enrichment analysis from gene expression data. Use analysis of variance to compare the cell subpopulations abundance in control, DM, and DMCHD.

## Results

### Determining DRAG Modules

**Figure 1:**
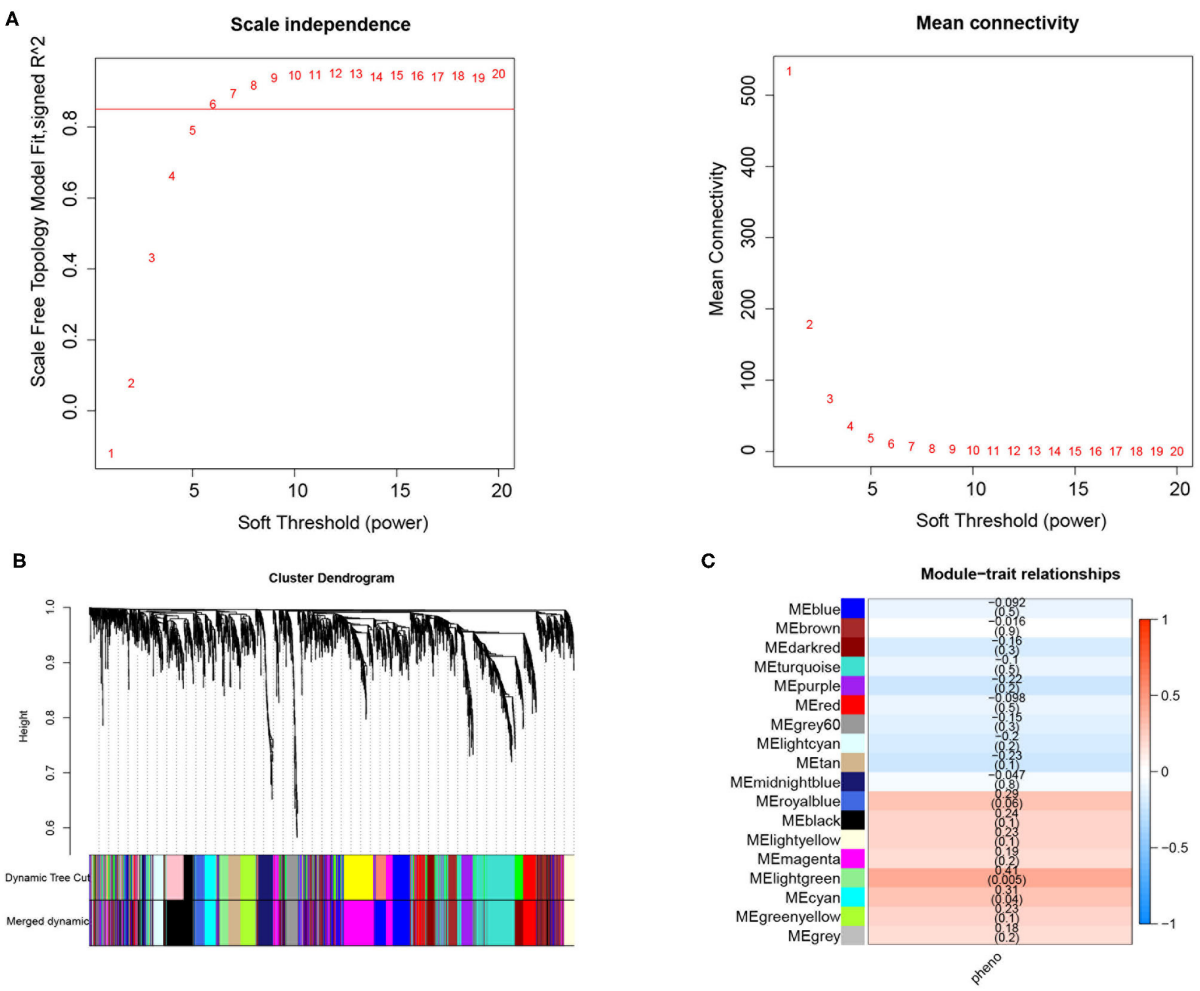
Weighted correlation network analysis to obtained the module associated with the development of diabetes mellitus-related atherogenesis in 33 healthy control samples, 15 DM samples and 15 DMCHD samples. **(A)** Graphical representation showing the selection of soft-thresholding powers. **(B)** Hierarchical clustering dendogram. **(C)** Histogram showing module-trait correlations. Light green module was the modules which had the strongest correlation with diabetes mellitus-related atherogenesis. DM, diabetes mellitus. DMCHD, diabetes mellitus-induced coronary heart disease.

In the current study, a total of 2,442 DEGs were screened with 1,029 up-regulated and 1,413 down-regulated in in DM and DMCHD compared to healthy control. The expression profiles of the 2,442 DEGs were subjected to WGCNA. In the WGCNA, according to the results of the scale-free fit index and average connectivity, the soft-thresholding was determined as six (Figure 1A). Based on hierarchical clustering, the genes were divided into 18 modules (Figure 1B). The light green module was involved in immune- and T cell activity-related biological processes, and displayed the most significant positive correlation between modules and DM-related atherogenesis (*r* = 0.41, *p* = 0.005) (Figure 1C). It was therefore considered as the candidate module in subsequent analyses.

### Biological Functions Involving the Candidate Module

**Figure 2:**
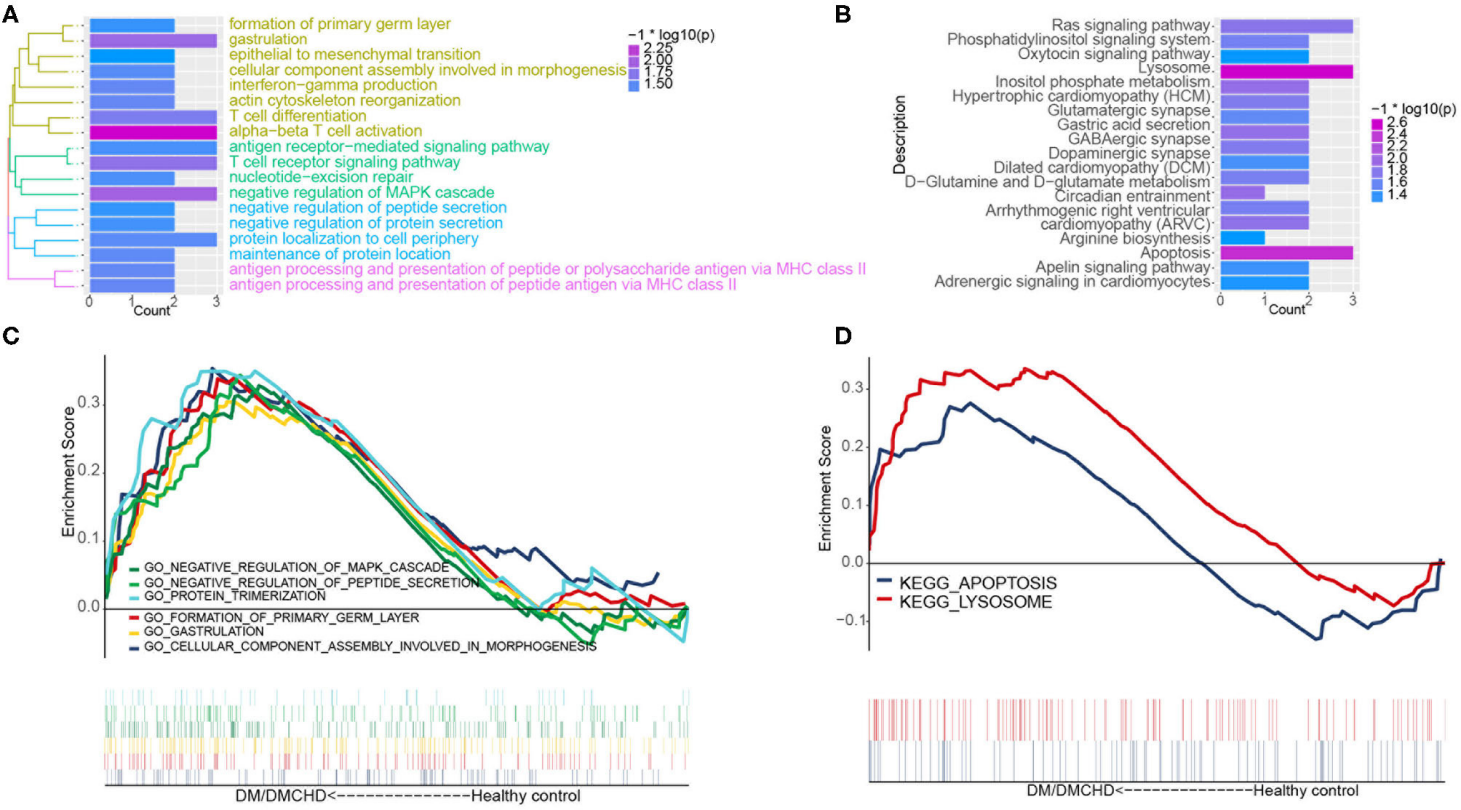
The exploration of DM and DMCHD-related biological process and pathways using functional enrichment analysis. **(A)** Significantly enriched biological processes corresponding to genes in the light green module. *P* < 0.05 was considered significant. **(B)** Significant enrichment of KEGG pathways for genes in the light green module. *P* < 0.05 was considered significant. **(C)** Biological processes identified by gene set enrichment analysis and found to be significantly enriched in samples from patients with diabetes mellitus or both diabetes mellitus (DM) and coronary heart disease (DMCHD). KEGG, Kyoto Encyclopedia of Genes and Genomes. **(D)** Biological processes determined from gene clusters. DM, diabetes mellitus; DMCHD, diabetes mellitus and coronary heart disease.

The light green module contained genes significantly involved in biological processes related to cytoskeletal anchoring at plasma membrane, protein localization to cell cortex and protein deneddylation (Figure 2A). Genes within the module were associated with autophagy, phosphatidylinositol biosynthetic processes and G protein-coupled receptor signaling pathways (Supplementary Figure 1). Moreover, the light green module was significantly involved in pathways involving lysosomes, apoptosis, Ras signaling pathways and inositol phosphate metabolism (Figure 2B). DM and DMCHD samples showed enrichment in gastrulation and protein trimerization (Figure 2C), as well as lysosome and apoptosis pathways (Figure 2D).

### Interaction Between Proteins Encoded by DM and CHD

**Figure 3:**
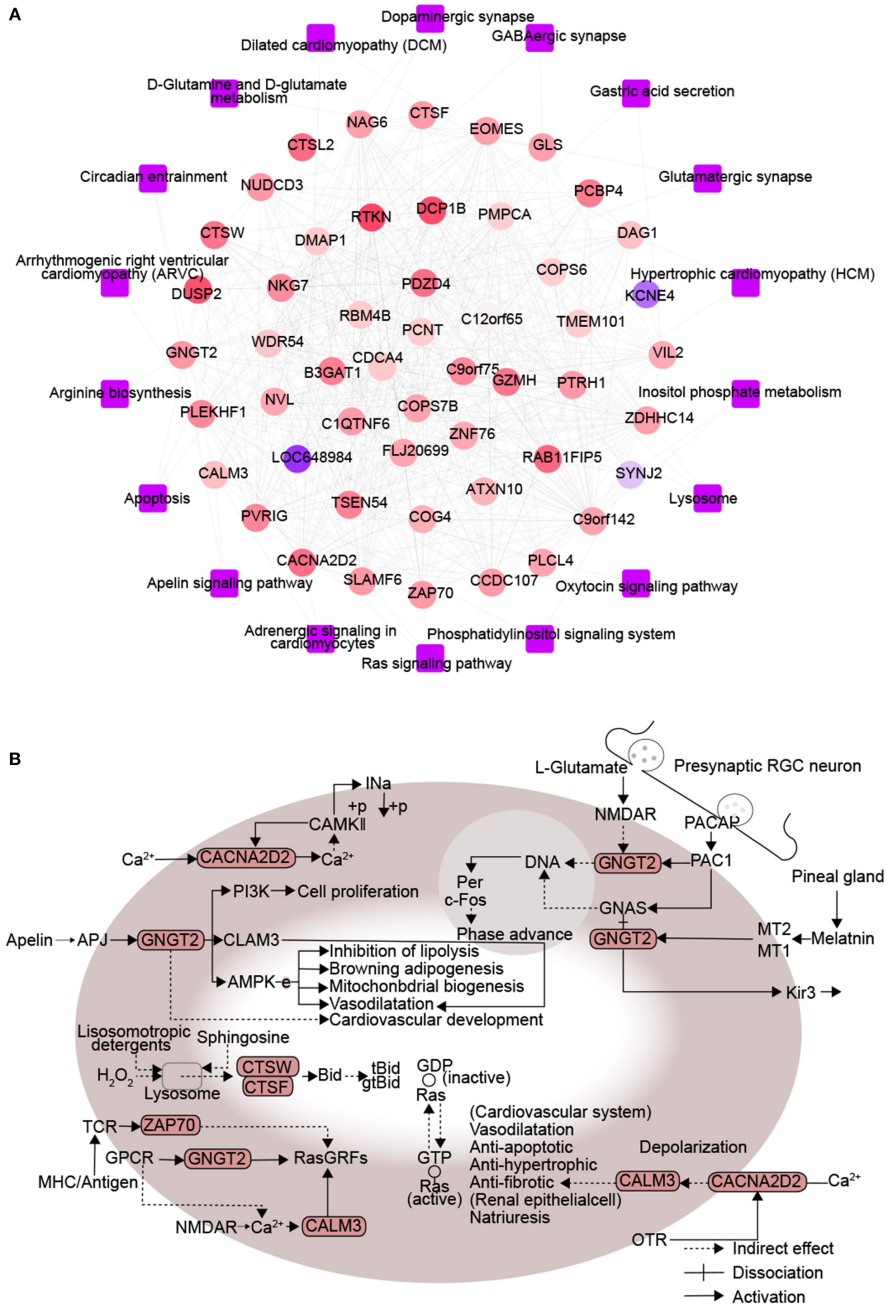
Protein interactions associated with signaling pathways enriched in diabetes mellitus-associated coronary heart disease. **(A)** Network diagram of protein-protein-KEGG pathway related to DMCAD. The ellipse for the module gene. The round rectangle for the KEGG pathway. The color of gene nodes represents MM, and the color at the edge of gene nodes represents modules. **(B)** CALM3, ZAP70, GNGT2, CACNA2D2, CTSF, and CTSW-related KEGG pathways. DMCHD, diabetes mellitus-induced coronary heart disease. DRAG, DM-related atherogenesis gene. MM, module membership.

A protein-protein KEGG pathway network was constructed to provide a potential mechanism explaining how DM increases risk of CHD. The network included 51 module-related genes and 18 KEGG pathways (Figure 3A). Previous studies proposed that arginine biosynthesis, GABAergic synapse and gastric acid secretion pathways overlap between DM and CHD (30–32). In this study, we elucidated the following pathways potentially involved in DMCHD: adrenergic signaling in cardiomyocytes, apelin signaling, circadian entrainment, oxytocin signaling pathway, apoptosis and Ras signaling (Figure 3B). The arenergic signaling in cardiomyocytes was activated by Ca^2+^ signaling, cell phosphorylation was mediated by CAMKII and CACNA2D2. In addition, we found that CNGT2 could affect cell proliferation. The apelin signaling pathway might cause vasodilatation and cardiovascular development. The G protein-coupled receptor APJ and its cognate ligand what apelin were widely expressed throughout the human body. They implicated in different key physiological processes such as angiogenesis, cardiovascular functions and energy metabolism regulation. Moreover, they also involved in the development and progression of different pathologies including diabetes, obesity and cardiovascular disease (33). In the circadian entrainment, presynaptic RGC neuron secreted L-glutamate and PACAP. Furthermore, PAC1 could activate c-Fos by CNGT2. In the oxytocin signaling pathway, CACNA2D2 and CALM3 could indirectly affect cardiovascular system and renal epithelial cell. Besides, the oxytocin signaling pathway was also activated by Ca2+ signaling. In the apoptosis, sphingosine and lysosomotropic detergents and H2O2 could indirectly affect lysosome. CTSW and CTSF could be indirectly affected by lysosome, thereby affecting cell apoptosis. In the Ras signaling pathway, CNGT2 and CALM3 could activate GDP-Ras inactive to GTP-Ras active process by RasGRFs. Moreover, ZAP70 could also indirectly affect the process by RasGRFs.

### The Expression of DRAGs and ROC Analysis of DM-Related Atherogenesis GSVA Score

**Figure 4:**
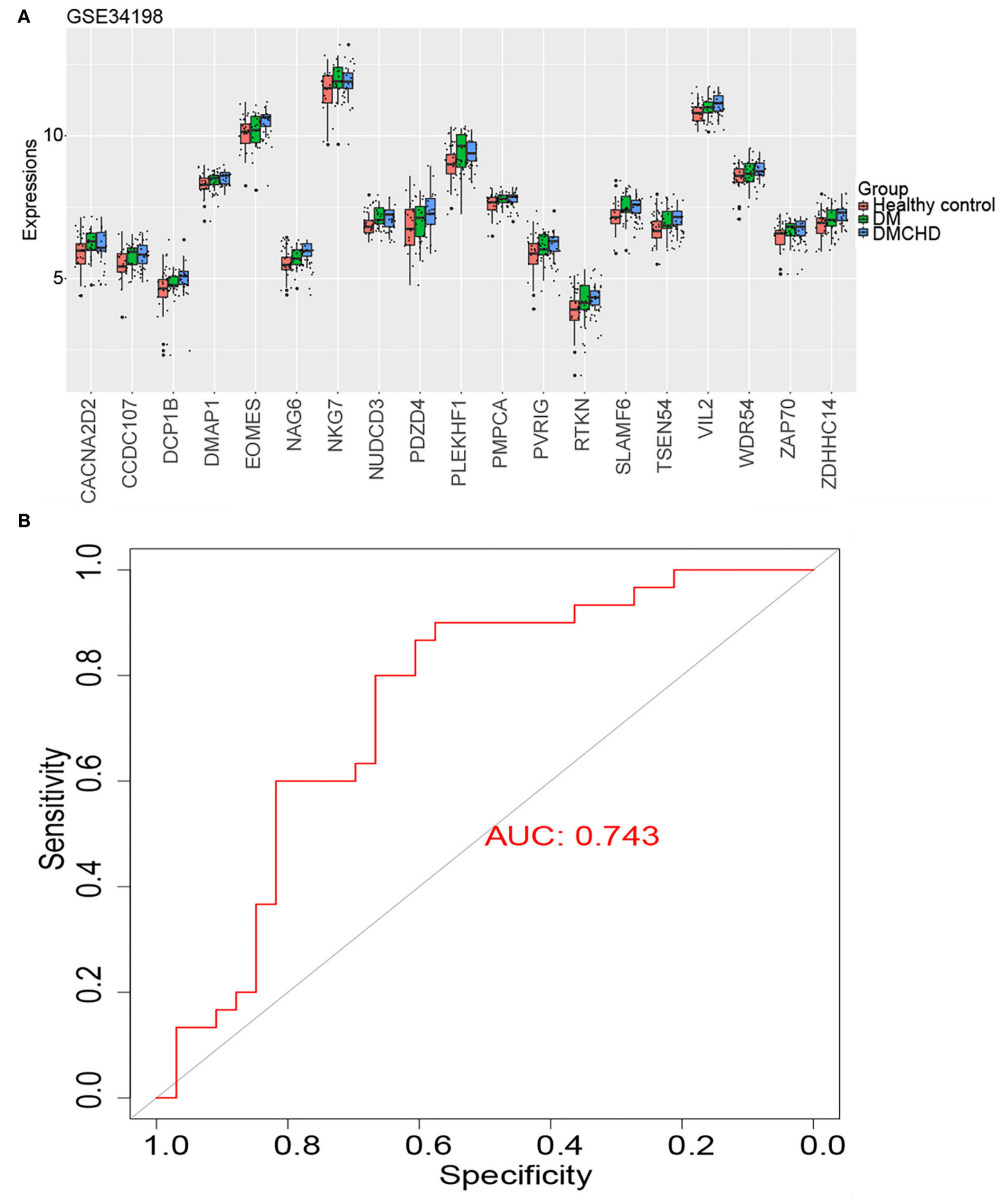
Expression of diabetes mellitus-related atherogenesis genes (DRAGs) and their ability to predict diabetes mellitus-associated coronary heart disease (DMCHD). **(A)** The expression level of 19 DRAGs (genes with significance (GS) > 0.2 and module membership (MM) > 0.7 were considered as DRAGs) in tissue from healthy individuals and from patients with diabetes mellitus (DM) or DMCHD. **(B)** Receiver operating characteristic curve analysis of DM-related atherogenesis GSVA score for predicting DMCHD.

We identified the following genes from the light green module as significant DRAG candidates: *RTKN, DCP1B, PDZD4, CACNA2D2, TSEN54, PVRIG, PLEKHF1, NKG7, ZAP70, NUDCD3, SLAMF6, CCDC107, NAG6, ZDHHC14, EOMES, VIL2, WDR54, DMAP1*, and *PMPCA*. Their expression progressively increased in healthy control, DM and DMCHD (Figure 4A), the differential expression of these genes in three different groups healthy control, DM, DMCHD might be the reason which lead to DM patients with coronary heart disease, but whether this is the cause of this result needs further exploration. ROC curves showed that the DM-related atherogenesis GSVA score can predict DMCHD, with an area under the ROC curve of 0.743 (Figure 4B).

### Validating Aberrant Expression of DRAGs

**Figure 5:**
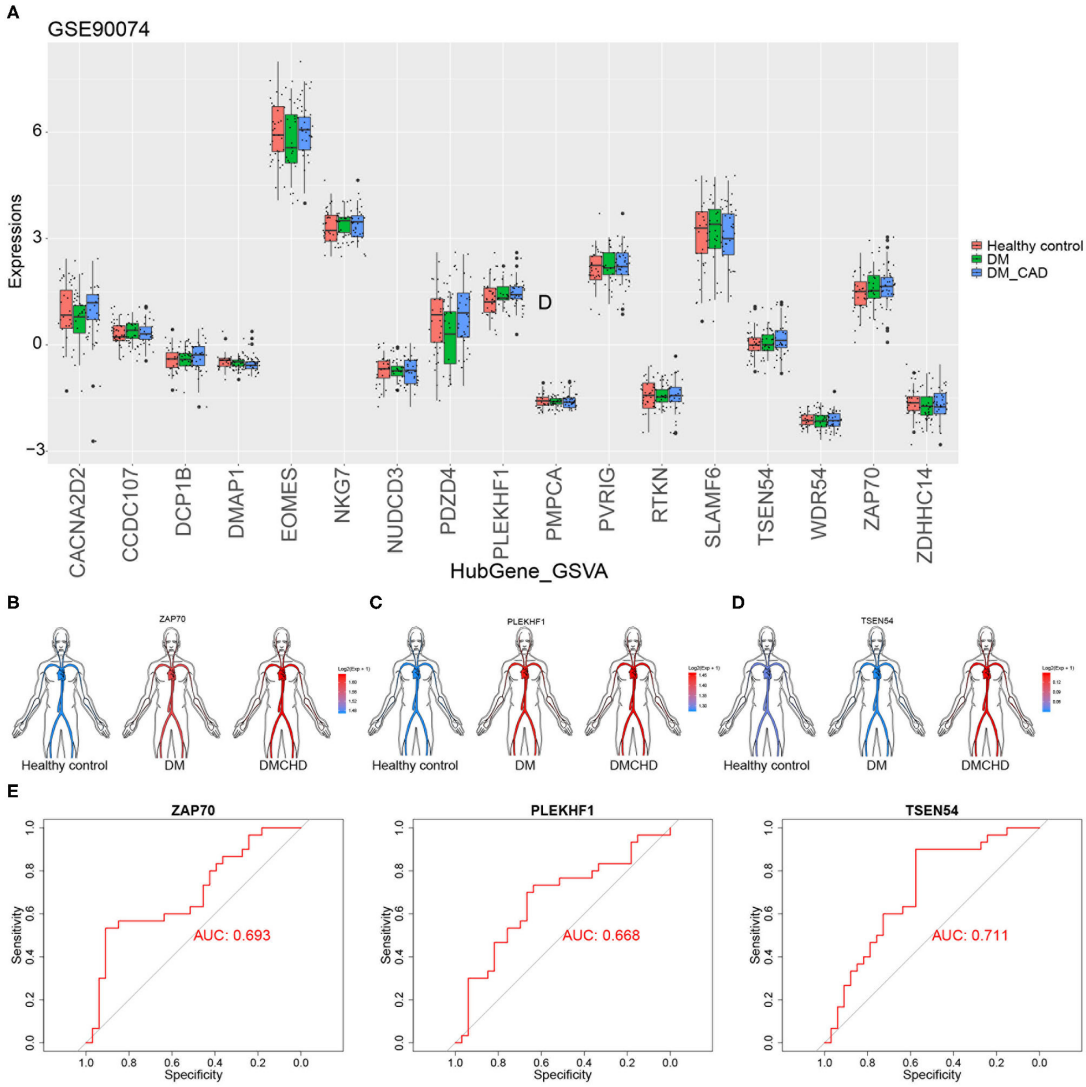
Validation of diabetes mellitus-related atherogenesis genes (DRAGs). **(A)** The expression of DRAGs in tissue samples from healthy controls or patients with diabetes mellitus (DM) or both diabetes mellitus and coronary heart disease (DMCHD) in a validation data set (GSE90074). Expression levels in GSE34198 of **(B)** *ZAP70*, **(C)** *PLEKHF1*, and **(D)** *TSEN54* in human circulation (https://github.com/jespermaag/gganatogram). **(E)** Receiver operating characteristic curves of *ZAP70, PLEKHF1* and *TSEN54* as potential biomarkers for predicting coronary heart disease in patients with diabetes mellitus.

To validate the DRAGs we identified, we examined their expression levels in the GSE90074 dataset. We confirmed that *ZAP70, PLEKHF1*, and *TSEN54* expression significantly progressively increased in healthy control, DM and DMCHD (Figures 5A–D), these genes were considered to be the progression genes of DM complicated with CHD. ROC curve analysis suggested that all three genes may be potential biomarkers of CHD development in DM (Figure 5E).

### Various Cell Types Vary in Control, DM and DMCHD

**Figure 6:**
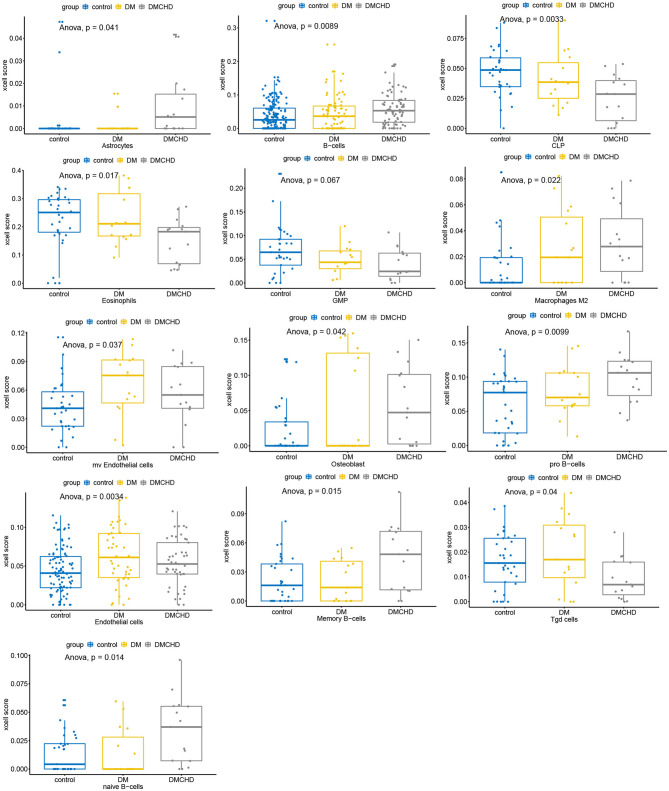
Various cell types vary in control, DM and DMCHD. DM, diabetes mellitus, DMCHD, diabetes mellitus and coronary heart disease, CLP, common lymphoid progenitor cell; GMP, granulocyte macrophage progenitor; Tgd cells, gamma delta T cells; mv endothelial cells, mitral valve endothelial cells. Anova, analysis of variance.

After estimating and comparing the cell subpopulation abundance, we found the various cell types vary in control, DM and DMCHD. B cells and Macrophages (M2) showed an increasing trend, while eosinophils, common lymphoid progenitor cell (CLP), and granulocyte macrophage progenitor (GMP) showed a downward trend. Osteoblast, Gamma delta T cells (Tgd cells), and endothelial cells are also different in control, DM and DMCHD (Figure 6).

## Discussion

DM is a syndrome characterized by chronic hyperglycemia accompanied by disorder of lipid and protein metabolism. The pathogenesis of diabetes includes insulin resistance and insulin secretion disorders of islet beta cells. CHD is a kind of heart disease caused by atherosclerotic lesion of coronary artery, which leads to stenosis or obstruction of vascular lumen and myocardial ischemia, hypoxia or necrosis. DM and CHD are common chronic diseases (34). People with DM have a much higher incidence of CHD because of dyslipidemia and metabolic disorders, particularly in the formation of glycated proteins, causing inflammation and artery stiffening as well as dysregulating hormones, immune responses and dendritic cell activity (35). However, the genetic changes that underlie development of CHD in patients with DM is not fully understood. In the present study, we performed WGCNA in a whole-blood gene expression profile to screen for gene modules involved in the pathogenesis of DM-related atherogenesis. We found that a light green module, a cluster of immune system genes, had the strongest correlation with emergence of CHD in DM. Gene sets associated with apoptosis and lysosomes were particularly enriched in DM and DMCHD samples. These findings are consistent with a known link between apoptosis and CHD (36) and between lysosome activity and DMCHD (37).

A protein-protein KEGG pathway network was constructed to help reveal the molecular mechanism of DMCHD. Among the six pathways identified, previous work has already implicated apelin signaling (38), apoptosis (39), and Ras signaling (40) in DMCHD. In addition, our study suggests that adrenergic signaling in cardiomyocytes, circadian entrainment and oxytocin signaling pathway are also related to DMCHD. *CACNA2D2* and *GNGT2* play important roles in regulating these pathways. Expression of *CACNA2D2* in the adrenergic signaling pathway elevates intracellular Ca^2+^ in cardiomyocytes, enhancing myocardial contractility (41), and more generally changes in gene expression have been associated with Ca^2+^ mobilization in CHD (42). Regulation of *GNGT2* affects circadian entrainment, in which c-Fos may be activated to regulate apoptosis, although this is controversial (43, 44). The activation of c-Fos in gestational diabetes suggests a link to glucose metabolism, which may therefore be linked to DMCHD, which requires further study. Notably, oxytocin promotes glucose metabolism in cultured cardiomyocytes from newborn and adult rats (45). Furthermore, it can directly improve pancreatic functions (46), presumably by increasing insulin early (47). Oxytocin signaling pathway is also activated by Ca^2+^ signaling in a *CACNA2D2*-dependent manner. These data support targeting oxytocin in DM patients in order to prevent CHD.

DM is a complicated, polygenic disease that exerts pleotropic effects that may increase risk of other diseases, such as CHD. Therefore, it is necessary to identify hallmark genes that explain potential mechanism of DM-related atherogenesis. We identified 19 genes in the light green module as DRAGs (*RTKN, DCP1B, PDZD4, CACNA2D2, TSEN54, PVRIG, PLEKHF1, NKG7, ZAP70, NUDCD3, SLAMF6, CCDC107, NAG6, ZDHHC14, EOMES, VIL2, WDR54, DMAP1*, and *PMPCA*), the differential expression of these genes in three different groups healthy control, DM, DMCHD might be the reason which lead to DM patients with coronary heart disease, but whether this is the cause of this result needs further exploration. We found that the DM-related atherogenesis GSVA score progressively increased in healthy control, DM and DMCHD samples. ROC analysis showed that DM-related atherogenesis GSVA score may have predictive potential for identifying genes responsible for DMCHD development. Our work suggests that the DRAGs *ZAP70, PLEKHF1*, and *TSEN54* may be biomarkers for CHD in DM patients. In addition, we also found some types of cells in whole blood showed upward or downward trend in control, DM and DMCHD. However, the abundance of the cell subsets will be affected by many factors, and whether it can be used as a specific marker of DM or DMCHD still needs further exploration.

There are several notable limitations in our study. First, we took a bioinformatics approach to mine potential molecular mechanisms causing DMCHD. Our findings will need to be followed up with mechanistic studies. Second, using GSVA to assess presence of genetic risk factors in individual patients is cost-prohibitive and therefore not applicable in clinics yet. Third, only one dataset was used for validation. Whether *ZAP70, PLEKHF1*, and *TSEN54* are true biomarkers for DMCHD requires further examination in additional, larger data sets. In addition, it is worth noting that the whole blood gene expression profiles were used in the present study. We also found various cell types vary in control, DM and DMCHD, however, we can not elaborate on the interactions between cells in the DM-related atherogenesis in the present study.

In conclusion, we identified 19 hallmark genes of DM-related atherogenesis, which might be the biomarkers for identifying DM and CHD. The DM-related atherogenesis GSVA score may predict susceptibility of DM patients to developing CHD. Our protein-protein KEGG pathway network may reveal molecular mechanisms underlying DMCHD development. These results provide numerous leads for future studies of CHD risk factors in DM, and may provide a theoretical basis for the treatment of DM and CHD patients.

## Data Availability Statement

The datasets generated for this study can be found in online repositories. The names of the repository/repositories and accession number(s) can be found in the article/Supplementary Material.

## Author Contributions

All authors participated in the design, interpretation of the studies, analysis of the data, and review of the manuscript. QH and GD conducted the experiments. RW supplied critical reagents. DZ wrote the manuscript. JW contributed to critically revise the manuscript. All authors contributed to the article and approved the submitted version.

## Conflict of Interest

The authors declare that the research was conducted in the absence of any commercial or financial relationships that could be construed as a potential conflict of interest.

## Supplementary Material

Supplementary Figure 1 Biological process enrichment analysis using *ClueGO* plugin for genes in the light green module.
