# Supplementary figures and images for: Comprehensive Identification of Key Genes Involved in Development of Diabetes Mellitus-Related Atherogenesis Using Weighted Gene Correlation Network Analysis
Source: Front Cardiovasc Med. 2020 Oct 28;7:580573. doi: 10.3389/fcvm.2020.580573 (PMC7655645; doi:10.3389/fcvm.2020.580573)

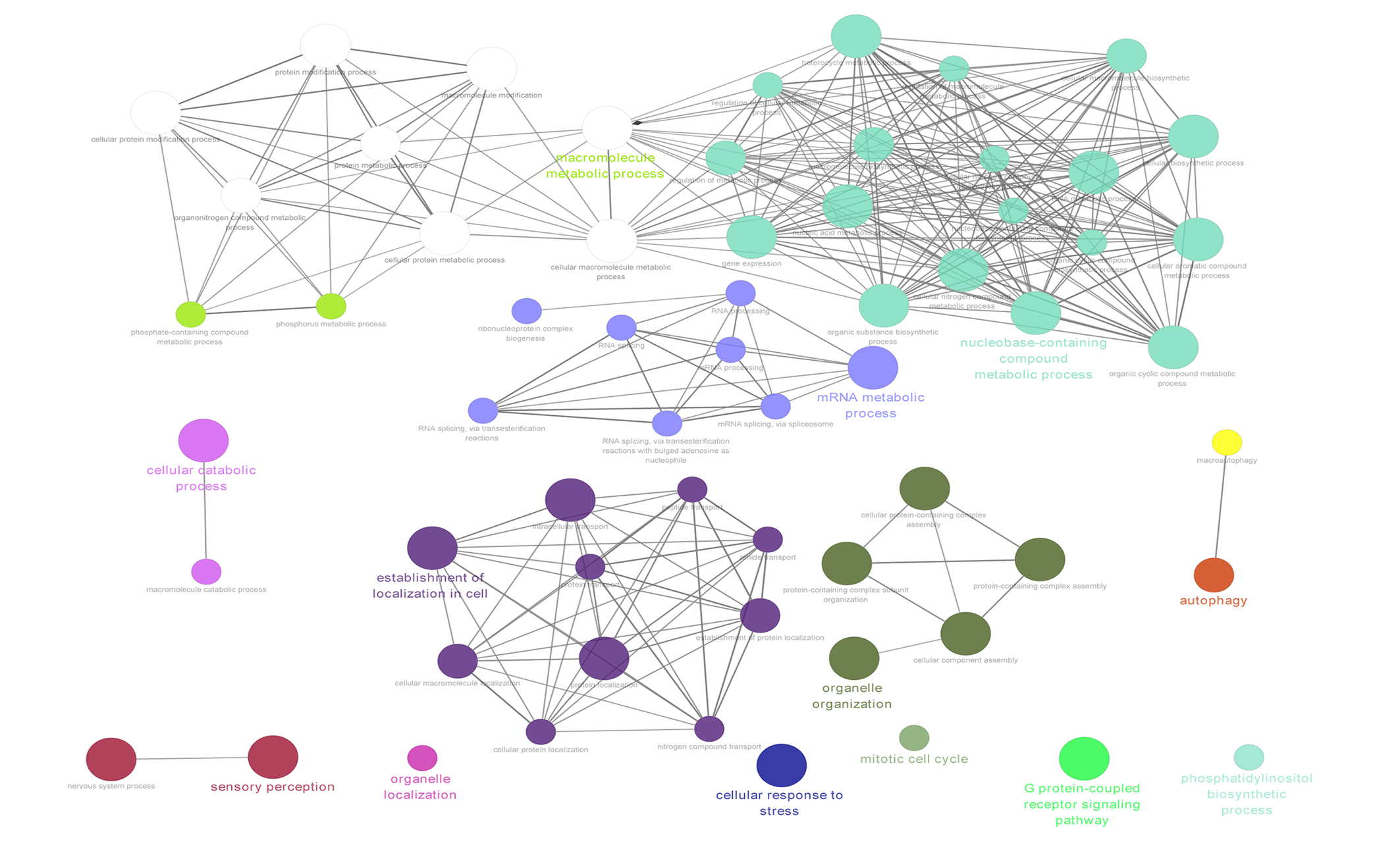

Supplement: Supplementary Figure 1 — Biological process enrichment analysis using ClueGO plugin for genes in the light green module. [file Image_1.TIF]
